# Comparison of a Novel MRNA-Based Breast Cancer Subtyping Assay with PAM50 and Oncotype DX in Estrogen Receptor-Positive/HER2-Negative Breast Cancer: An Exploratory Age-Stratified Retrospective Cohort Study

**DOI:** 10.3390/cimb48090886

**Published:** 2026-08-31

**Authors:** Till Wallrabenstein, Elena Diana Chiru, Martina Sonderegger, Simone Muenst, Christian Kurzeder, Marcus Vetter

**Affiliations:** 1 Center for Oncology and Hematology, Cantonal Hospital Baselland, 4410 Liestal, Switzerland; elena-diana.chiru@ksbl.ch (E.D.C.); martina.sonderegger@ksbl.ch (M.S.); marcus.vetter@ksbl.ch (M.V.); 2 Institute of Pathology and Medical Genetics, University Hospital Basel, 4031 Basel, Switzerland; 3 Breast Clinic, University Hospital Basel, 4031 Basel, Switzerland; 4 Breast Center Baselland, Cantonal Hospital Baselland, 4410 Liestal, Switzerland; 5 Cancer Center Baselland, Cantonal Hospital Baselland, 4410 Liestal, Switzerland; 6 Medical Faculty, University of Basel, 4001 Basel, Switzerland

**Keywords:** breast cancer, BCSK, gene expression profiling, molecular subtyping, PAM50, Oncotype DX, proliferation, recurrence risk

## Abstract

Immunohistochemistry (IHC) of ER, PR, HER2, and Ki67 are established prognostic and predictive markers in breast cancer. Gene expression assays such as PAM50 and Oncotype DX are increasingly used for molecular subtyping and recurrence risk calculation. The APIS Breast Cancer Subtyping Kit (BCSK) quantitatively measures the mRNA expression of these markers (ER, PR, HER2, and Ki67) and includes a novel four-gene BCSK Proliferation Signature (PS) designed for subtype classification and risk stratification. This exploratory retrospective cohort study compares the BCSK with established molecular assays in an age-stratified cohort. We aimed to compare BCSK subtype classification in distinguishing Luminal A versus Luminal B subtypes with IHC and with PAM50. We have also aimed to assess correlations between the BCSK PS and the Oncotype DX Recurrence Score (RS) as well as the PAM50 Risk of Recurrence (ROR) score in patients stratified by age (<65 versus ≥65 years). Formalin-fixed, paraffin-embedded (FFPE) tumor specimens from 59 patients with ER+/HER2− breast cancer (33 < 65 years, 26 ≥ 65 years), diagnosed between 2020 and 2022 at the Cantonal Hospital Baselland and University Hospital Basel, were analyzed using IHC, BCSK, and PAM50 (Prosigna^®^). All patients received adjuvant therapy and had ODx scores available. Patient, disease and treatment characteristics were compared between age groups using the Fisher exact test. We assessed concordance in luminal subtype classification across IHC, BCSK, and PAM50 assays pairwise and stratified by age groups descriptively. We used Spearman’s rank correlation to examine associations between BCSK PS, RS, and ROR. Differences between age groups regarding PS were analyzed using the Mann–Whitney U test. In the ≥65-year cohort, subtype classification concordance between the BCSK and PAM50 was higher (84.6%) than in those aged <65-years (60.6%). In patients aged <65 years, the PS was significantly correlated with both RS (ρ = 0.5745, *p* < 0.0005) and ROR (ρ = 0.391, *p* = 0.024), whereas RS and ROR were not significantly correlated. In patients ≥65 years, PS correlated significantly with ROR (ρ = 0.603, *p* = 0.001), while there were no significant correlations between PS and RS (ρ = 0.134, *p* = 0.514) and between RS and ROR (ρ = 0.149, *p* = 0.466). Median PS values did not significantly differ between age groups (0.589 versus 0.602, *p* = 0.97). In this exploratory retrospective age-stratified analysis, the BCSK demonstrated descriptive concordance with PAM50 subtype classifications, especially in patients aged ≥65 years. PS showed significant but moderate correlation with the established molecular recurrence scores RS and ROR, however these differed across age groups. Our findings should be considered hypothesis-generating because this study did not include outcome metrics and was based on a relatively small cohort. Larger prospective studies incorporating clinical outcomes are necessary to determine the diagnostic and prognostic utility as well as comparative cost-effectiveness of the BCSK and its utility across age groups.

## 1. Introduction

Breast cancer (BC) is the most diagnosed cancer in women worldwide and represents a significant public health challenge [1]. Most cases fall within the hormone receptor-positive (HR+) and HER2-negative subgroup, characterized by the expression of estrogen (ER) and/or progesterone (PR) receptors in the absence of HER2 overexpression [2]. Within this subgroup, tumors are further classified into Luminal A and Luminal B molecular subtypes, each associated with distinct biological characteristics, therapeutic responses, and clinical outcomes [3]. Luminal A tumors typically have lower proliferation rates, higher expression of hormone receptors, and are associated with a more favorable prognosis. In contrast, Luminal B tumors often display higher proliferation indices, lower hormone receptor expression, and a comparatively higher risk of recurrence [3,4,5,6].

Traditional classification of these subtypes relies on immunohistochemical (IHC) analyses of markers such as ER, PR, HER2, and Ki67 [4,5]. However, IHC-based assessments are subject to inter-observer variability and differences in laboratory protocols can affect consistency and reproducibility [7,8,9]. Additionally, the distinction between Luminal A and B subtypes using the Ki67 proliferation marker is particularly challenging due to the lack of consensus on cut-off values and variable expression levels. This has led to the growing adoption of molecular diagnostic tools that offer quantitative assessments and can be easily standardized [10,11,12].

Assays such as PAM50 (Prosigna^®^) and Oncotype DX are now widely used to provide molecular subtype classification and risk stratification. PAM50 utilizes gene expression profiling to classify tumors into intrinsic subtypes, including Luminal A, Luminal B, HER2-enriched, and basal-like, and to calculate a Risk of Recurrence (ROR) score [13,14,15,16,17,18]. Oncotype DX (ODx), by contrast, analyzes the expression of 21 genes to produce a Recurrence Score (RS), which estimates the risk of distant recurrence and the potential benefit of chemotherapy in HR+/HER2− early breast cancer [11,14]. Although both assays are clinically validated and recommended in treatment guidelines, their cost and restricted availability in some settings underscore the need for alternative molecular testing options [13].

The APIS Breast Cancer Subtyping Kit (BCSK) is a novel molecular assay developed to measure mRNA expression levels of ER, PR, HER2, and Ki67 (based on expression of mRNA from the corresponding genes ESR1, PGR, ERBB2, and MKI67), along with a proprietary four-gene Proliferative Signature (PS), which is based on mRNA expression levels of MKI67 and three additional genes (CCNA2, PCNA, and KIF23) [19,20]. This kit is intended to provide molecular subtype classification and proliferation-related information within a single platform of focused gene expression analysis, pending clinical validation. By generating objective and quantitative results, the BCSK may address some of the limitations of IHC-based classification and seeks to provide an alternative focused gene-expression approach [19,20].

The overall concordance of the BCSK subtype classification compared with IHC and PAM50 in a real-world cohort of 59 patients with HR+/HER2− BC has recently been reported [21]. In this exploratory retrospective study, we assess age-specific differences of the BCSK regarding luminal subtype differentiation. Results are compared with those from IHC, PAM50, and ODx. The aim of this age-stratified subgroup analysis is to examine potential age-related variations in BCSK assay performance. Given the rising incidence of BC in older patients and the increasing role of geriatric oncology in clinical decision-making, we have done a predefined exploratory age-stratified analysis using 65 years as the a priori threshold.

## 2. Materials and Methods

### 2.1. Patients

This study included all patients who had histologically confirmed hormone receptor-positive, HER2-negative invasive early breast cancer, have received surgical resection between January 2020 and December 2022 at the University Hospital Basel or the Cantonal Hospital Baselland, had formalin-fixed paraffin-embedded (FFPE) tumor tissue available for molecular analysis, and had complete immunohistochemical, Oncotype Dx, and Pam50 results. Patients were excluded if adequate tumor material was unavailable, molecular assay results were incomplete, or consent for tissue and data use for scientific purposes was denied. All individuals had undergone adjuvant endocrine therapy.

Patients were stratified by age into two cohorts: <65 years (n = 33) and ≥65 years (n = 26). This cut-off was selected a priori for the predefined exploratory age-stratified analysis and was not intended to represent a biologically defined threshold. This threshold was also close to the median age of our population, rendering two similarly sized groups.

### 2.2. Breast Cancer Subtyping Kit (Apis Score^®^)

BCSK quantifies the relative expression of four proliferation-associated genes (MKI67, CCNA2, PCNA, and KIF23) using RT–qPCR, and applies a logistic regression model to derive a continuous Proliferation Score (PS) ranging from 0 to 1. Scores < 0.5 were classified as low proliferation, while scores ≥ 0.5 indicated high proliferative activity [20].

Molecular analyses were performed centrally at Propath UK Ltd., Hereford, UK. RNA was extracted from FFPE tissue sections using the QIAGEN DSP RNeasy FFPE Kit (Qiagen GmbH, Hilden, Germany, cat. no. 73604) and was then analyzed using the APIS Breast Cancer Subtyping Kit (Apis Assay Technologies Ltd., Manchester, UK) according to the manufacturer’s validated protocol. The assay quantifies mRNA expression of ESR1, PGR, ERBB2, MKI67, CCNA2, PCNA and KIF23 by RT-qPCR from FFPE tissue. Expression values were normalized using the reference genes IPO8 and PUM1 and reported as ΔCt values calculated from duplicate measurements. Positive and negative assay controls were included in each analytical run, and molecular subtype classification together with the BCSK Proliferation Score were generated using the proprietary APIS analysis software (version 1.2.1). Further details regarding assay methodology, analytical performance, and clinical implementation have been published previously [19,20,21].

### 2.3. Analytical Methods Regarding Immunohistochemistry, Oncotype and Pam50

ODx RS was determined from resection specimens submitted to Exact Sciences (Genomic Health, Redwood City, CA, USA), with results extracted from original pathology reports and interpreted according to the manufacturer’s guidelines. In accordance with the TAILORx trial criteria, scores ≥ 26 were classified as high risk in patients aged >50 years. For patients aged <50 years, scores of 21–25 were considered intermediate risk; all other scores were categorized as low risk.

Intrinsic subtypes and Risk of Recurrence (ROR) scores were available for all patients using the Prosigna PAM50 assay, performed on the nCounter Analysis System (NanoString Technologies, Seattle, WA, USA) with the PAM50 Codeset, according to the manufacturer’s protocol [22,23]. All analyses were conducted at a central reference laboratory (Propath UK Limited, Hereford, UK). Tumors were classified into intrinsic subtypes—Luminal A, Luminal B, HER2-enriched, or basal-like—based on PAM50 results. No cases were excluded on the basis of intrinsic subtype assignment discordant to IHC. Tumors classified as basal-like or HER2-enriched by PAM50 were retained in the analyses because intrinsic molecular subtype classification is not directly equivalent to surrogate immunohistochemical subtype classification. ROR scores ≤ 40 were defined as low risk, scores of 41–60 as intermediate risk, and scores > 60 as high risk.

Eligibility for inclusion in the study cohort was determined based on IHC profiling (ER, PR, HER2, and Ki67) performed on diagnostic core needle biopsies at the Institute of Pathology, University Hospital Basel, and the Cantonal Hospital Baselland, Switzerland. For all 59 cases undergoing PAM50 assessment, Ki67 immunostaining was repeated on resection specimens to account for potential changes since the initial biopsy. IHC was performed on FFPE sections by Source BioScience (Nottingham, UK). In samples classified as basal-like or HER2-enriched by PAM50, repeat staining for ER, PR, and HER2 was also conducted. All IHC analyses were performed using the Ventana Benchmark Ultra platform with Ventana assays (F. Hoffmann-La Roche Ltd., Basel, Switzerland): ER (clone SP1), PR (clone 1E2), Ki67 (clone 30-9), and HER2 (clone 4B5), according to the manufacturer’s protocols. IHC-based molecular subtypes were assigned following the European Society for Medical Oncology (ESMO) clinical guidelines [24]. Immunostains were evaluated in accordance with ASCO/CAP guidelines [25,26]. Ki67 status was defined as positive when nuclear staining was observed in >20% of tumor cells, in accordance with local practice standards. 

Subtype assignments generated by IHC, BCSK, and PAM50 were recorded independently. No adjudication procedure was performed for discordant classifications, as the objective of the study was to compare molecular subtype assignments between the different methods rather than to establish a reference diagnosis. All original assay results were therefore retained for analysis.

### 2.4. Statistical Methods

Concordance in luminal subtype classification across IHC, BCSK, and PAM50 was evaluated using descriptive statistics. Concordance was calculated pairwise and defined as percentage of patients equally classified by two respective methods. Associations between the BCSK Proliferation Score (PS), ODx Recurrence Score (RS), and PAM50 Risk of Recurrence (ROR) were assessed using Spearman’s rank correlation coefficient. Statistical comparisons of continuous variables between age groups were performed using the Mann–Whitney-U test. All tests were two-sided, and a *p*-value < 0.05 was considered indicative of statistical significance. Given the exploratory nature of this study and the lack of prior data on BCSK in age-stratified cohorts, no formal sample size calculation was performed. *p*-values were not adjusted for multiple comparisons and should therefore be interpreted accordingly.

## 3. Results

### 3.1. Patient Characteristics

We analyzed tumor specimens from 59 patients with ER-positive, HER2-negative breast cancer, stratified by age into two cohorts: 33 patients aged <65 years and 26 aged ≥65 years. Baseline, disease, and treatment characteristics are summarized in Table 1. As expected, significantly more patients were post-menopausal in the older age group and also relevant comorbidities tended to be more frequent. The distribution of the T-stage differed between groups with pTt3 tumors occurring only in the ≥65 years group. There were no significant differences between the two groups regarding the N-stage, expression of PR or Ki67, and grade, as well as the frequency of adjuvant chemo- and radiotherapy.

### 3.2. Subtype Classification Concordance

Molecular subtype classifications derived from IHC, the BCSK assay, and PAM50 revealed variable concordance as shown pairwise in Table 2. PAM50 also identified basal-like and HER2-enriched intrinsic subtypes within our IHC-defined ER+/HER2− cohort. These cases were retained for analysis.

Among patients <65 years (n = 33), IHC classified 15 (45.5%) as Luminal A and 18 (54.5%) as Luminal B HER2−. In this group BCSK classified 14 (42.4%) and 19 (57.6%) patients as Luminal A and B HER2−, respectively, with five patients being reclassified to Luminal B HER2− and four patients to Luminal A, resulting in overall concordance in 72.7% of patients between IHC and the BCSK. In this cohort, the BCSK and PAM50 agreed in 61% of cases. PAM50 identified 21 patients (63.6%) as Luminal A and nine (27.3%) as Luminal B HER2−, with nine of IHC-defined Luminal B HER2− cases reclassified as Luminal A.

In patients aged ≥65 years (n = 26), IHC classified 11 patients (42.3%) as Luminal A and 15 patients (57.7%) as Luminal B HER2−. In this group, the BCSK classified 13 patients each as Luminal A or as Luminal B/HER2− (each 50%), with three patients being reclassified to Luminal B/HER2− and five patients to Luminal A, resulting in overall concordance in 69% of patients between IHC and the BCSK. In this age group, PAM50 classified 14 patients (53.8%) as Luminal A and 11 (42.3%) as Luminal B HER2−. The BCSK and PAM50 matched in 85% of cases.

The comparison of IHC and PAM50 classifications rendered low overall concordance, however it was similar between age groups (54.5% in patients aged <65 and 53.8% in patients aged ≥65 years). Cross-platform concordance was also similar between age groups regarding IHC versus the BCSK (72.7% in patients aged <65 and 69.2% in patients aged ≥65 years). Interestingly, the cross-platform concordance for BCSK–PAM50 was descriptively higher in patients aged ≥65 years (84.6%) than in those aged <65 years (60.6%).

### 3.3. Correlation of BCSK Proliferation Score with Oncotype Dx and Pam50

Spearman correlation analyses were conducted to evaluate associations between the BCSK Proliferation Score (PS), Oncotype DX Recurrence Score (RS), and PAM50 Risk of Recurrence (ROR). The results are summarized in Table 3.

Among patients aged <65 years (n = 33), PS showed a moderate positive correlation with the Oncotype RS (ρ = 0.5745, *p* < 0.0005) and with the PAM50 ROR score (ρ = 0.391, *p* = 0.024), while RS and ROR showed a weaker, non-significant correlation (ρ = 0.242, *p* = 0.175).

In patients aged ≥65 years (n = 26), PS showed a significant positive correlation with PAM50 ROR (ρ = 0.603, *p* = 0.001), whereas there was no significant correlation between PS and RS (ρ = 0.134, *p* = 0.514) nor between RS and ROR (ρ = 0.149, *p* = 0.466).

### 3.4. Comparison of BCSK Proliferation Score Between Age Groups

The median BCSK Proliferation Score (PS) was similar between patients aged <65 years (median, 0.589) and those aged ≥65 years (median, 0.602). No statistically significant difference in BCSK PS was observed between the two age groups (Mann–Whitney U = 426, *p* = 0.970).

## 4. Discussion

In this exploratory retrospective study, we evaluated the performance of the BCSK in classifying luminal subtypes as compared to IHC and PAM50 and compared the BCSK-derived Proliferation Score (PS) with Oncotype DX RS and PAM50 ROR in patients with HR+/HER2− early breast cancer, stratified by age (<65 vs. ≥65 years). Using parallel assessments with IHC, PAM50, and Oncotype DX, the BCSK demonstrated descriptive concordance with PAM50, particularly in older patients. The BCSK-derived PS showed significant correlations with both RS and ROR in patients aged <65 years and with ROR, but not RS, in patients aged ≥65 years. These findings demonstrate associations of the BCSK with established molecular classification and recurrence metrics but do not establish clinical prognostic performance. The clinical utility, practicability, and cost-effectiveness of the BCSK remain to be validated in larger prospective studies, including comparisons not only with PAM50 and Oncotype DX, but also with well-established immunohistochemistry (IHC)-based surrogate assays such as IHC4 or ESMO surrogate subtype criteria that have demonstrated variable correlation with PAM50 and Oncotype DX [18,27,28,29,30].

A notable observation was the descriptively higher concordance between the BCSK and PAM50 in patients aged ≥65 years (concordance in 84.6% of patients) relative to those <65 years (concordance in 60.6% of patients). However, given the exploratory design and small subgroup sizes, this difference must be interpreted cautiously, because no formal statistical comparison of concordance between age groups was performed. Whether this age-stratified difference reflects underlying differences in tumor biology, cohort characteristics, or methodological factors cannot be determined from this present study and needs confirmation in larger independent cohorts.

A small number of tumors classified as HR+/HER2− by immunohistochemistry were assigned Basal-like or HER2-enriched intrinsic subtypes by PAM50. This finding has previously been described and may reflect differences between intrinsic molecular and surrogate immunohistochemical subtype classification rather than analytical discordance. Such cases may contribute to reduced concordance between the different classification methods and should be considered when interpreting pairwise comparisons.

Another key observation was the correlation of PS with established molecular recurrence metrics, although the correlation differed between age groups. In patients aged <65 years, PS correlated significantly with both RS and ROR, whereas in patients aged ≥65 year, it correlated significantly only with ROR but not with RS. Notably, the correlation between RS and ROR was weak and non-significant in both cohorts, consistent with prior studies that have reported discordance between these assays [18]. The observed correlations suggest that PS may capture molecular features related to those of established recurrence scores.

No significant differences in the BCSK Proliferation Score were observed between age groups. Although concordance between assays differed descriptively across age strata, the exploratory nature and limited sample size preclude firm conclusions regarding age-dependent biological differences. From a clinical standpoint, the BCSK might offer several potential advantages. By aiming to integrate subtype classification and proliferation-related molecular information into a single compact mRNA-based assay, it might enable quantitative and standardized reporting that could reduce the subjective variability associated with IHC. Moreover, the potential cost-effectiveness and scalability of the BCSK compared with established molecular assays warrant evaluation in future studies, particularly with regards to its potential accessibility in resource-limited settings.

However, this study has several limitations. The study was exploratory with a small sample size underpowered for reliable multivariable modelling, reporting descriptive concordance between molecular assays rather than formal diagnostic agreement statistics. Also no adjustment for multiple testing was performed because the analyses were exploratory. Although key associations reached statistical significance, effect sizes were generally moderate. These associations should therefore be interpreted with caution, and larger prospective cohorts will be necessary to confirm these findings and establish generalizability across clinical variables such as tumor grade, stage, or treatment. The retrospective design and absence of long-term clinical outcome data preclude direct assessment of prognostic performance.

Accordingly, the independent and age-specific prognostic value of the BCSK PS remains to be evaluated through adequately powered outcome-linked studies including multivariate modeling for clinical and pathological variables beyond age.

## 5. Conclusions

The BCSK showed concordance with established molecular subtyping and its Proliferation Score was associated with established molecular recurrence metrics across age groups in HR+/HER2− breast cancer. These exploratory findings support further evaluation of the BCSK as a molecular classification tool. Its clinical and prognostic utility remain to be validated in larger prospective, outcome-linked studies, while its feasibility and cost-effectiveness in comparison to established molecular assays require dedicated evaluation.

## Figures and Tables

**Table 1:** Baseline, disease and treatment characteristics of 59 patients with HR+/HER2− BC divided into two groups according to age (<65 years and ≥65 years).

Variable	Total (n = 59)	Age < 65 Years (n = 33)	Age ≥ 65 Years (n = 26)	*p*-Value
Median age (range)	63	52 (30–64)	72 (65–80)	n.a.
Female (%)	57 (97)	31 (94)	26 (100)	0.499
Post-menopausal (%)	41 (69)	15 (45)	26 (100)	<0.001
Relevant comorbidity	18 (31)	7 (21)	11 (42)	0.096
pT-stage				0.033
pT1	21 (36)	12 (36)	9 (35)	
pT2	32 (54)	20 (61)	12 (46)	
pT3	5 (8)	0	5 (19)	
pT4	1 (2)	1 (3)	0	
pN-stage				0.181
pN0	37 (63)	18 (55)	19 (73)	
pN+	22 (37)	15 (45)	7 (27)	
PR+	52 (88)	31 (94)	21 (81)	0.223
Ki-67 > 20%	30 (51)	17 (52)	13 (50)	1
Tumor grade				1
1	2 (3)	1 (3)	1 (4)	
2	30 (51)	17 (52)	13 (50)	
3	27 (46)	15 (45)	12 (46)	
Adjuvant chemotherapy	17 (29)	12 (36)	5 (19)	0.247
Adjuvant radiotherapy	48 (81)	27 (82)	21 (81)	1

Abbreviations: BC, breast cancer; HER2, human epidermal growth factor receptor 2; HR, hormone receptor; Ki67, Ki-67 proliferation index; n.a., not applicable; pN, pathological nodal stage; PR, progesterone receptor; pT, pathological tumor stage.

**Table 2:** Comparison of luminal subtype classification across immunohistochemistry (IHC), APIS Breast Cancer Subtyping Kit (BCSK), and Prosigna PAM50 in patients Aged <65 and ≥65 Years.

**A. IHC (Reference) vs. APIS Breast Cancer Subtyping Kit (BCSK)**							
**<65 Years (n = 33)**				**≥65 Years (n = 26)**			
**IHC Subtype**	**n**	**BCSK Subtype**	**n**	**IHC Subtype**	**n**	**BCSK Subtype**	**n**
Luminal A	15	Luminal A	10	Luminal A	11	Luminal A	8
		Luminal B HER2−	5			Luminal B HER2−	3
Luminal B HER2−	18	Luminal A	4	Luminal B HER2−	15	Luminal A	5
		Luminal B HER2−	14			Luminal B HER2−	10
**B. IHC (Reference) vs. PAM50**							
**<65 Years (n = 33)**				**≥65 Years (n = 26)**			
**IHC Subtype**	**n**	**PAM50 Subtype**	**n**	**IHC Subtype**	**n**	**PAM50 Subtype**	**n**
Luminal A	15	Luminal A	12	Luminal A	11	Luminal A	7
		Luminal B HER2−	3			Luminal B HER2−	4
Luminal B HER2−	18	Luminal A	9	Luminal B HER2−	15	Luminal A	7
		Luminal B HER2−	6			Luminal B HER2−	7
		Basal-like	3			HER2-enriched	1
**C. PAM50 (Reference) vs. APIS Breast Cancer Subtyping Kit (BCSK)**							
**<65 Years (n = 33)**				**≥65 Years (n = 26)**			
**PAM50 Subtype**	**n**	**BCSK Subtype**	**n**	**PAM50 Subtype**	**n**	**BCSK Subtype**	**n**
Luminal A	21	Luminal A	12	Luminal A	14	Luminal A	12
		Luminal B HER2−	9			Luminal B HER2−	2
Luminal B HER2−	9	Luminal A	1	Luminal B HER2−	11	Luminal A	1
		Luminal B HER2−	8			Luminal B HER2−	10
Basal-like	3	Luminal A	1	HER2-enriched	1	Luminal B HER2−	1
		Luminal B HER2−	2				

Abbreviations: BCSK, Breast Cancer Subtyping Kit; ER, estrogen receptor; HER2, human epidermal growth factor receptor 2; IHC, immunohistochemistry; pN, pathological nodal stage; PR, progesterone receptor; pT, pathological tumor stage.

**Table 3:** Spearman rank correlations between the BCSK Proliferation Score, Oncotype DX Recurrence Score, and PAM50 Risk of Recurrence (ROR) score in patients aged <65 years and ≥65 years. Correlation coefficients (ρ) and *p*-values are presented for each age group.

Variable	by Variable	Patients Aged <65		Patients Aged ≥65	
		Spearman ρ	*p*	Spearman ρ	*p*
BCSK PS	Oncotype Dx RS	0.5745	<0.0005	0.1339	0.5144
Oncotype Dx RS	PAM50 ROR	0.2420	0.1749	0.1494	0.4664
PAM50 ROR	BCSK PS	0.3912	0.0244	0.6031	0.0011

Abbreviations: BCSK PS, Breast Cancer Subtyping Kit Proliferation Score; PAM50, Prediction Analysis of Microarray 50-gene signature; *p*, *p*-value; ROR, Risk of Recurrence; RS, Recurrence Score; ρ, Spearman rank correlation coefficient.

## Data Availability

The data presented in this study are available on request from the corresponding author due to ethical considerations.
